# Implication of RNA-Binding Protein La in Proliferation, Migration and Invasion of Lymph Node-Metastasized Hypopharyngeal SCC Cells

**DOI:** 10.1371/journal.pone.0025402

**Published:** 2011-10-10

**Authors:** Gunhild Sommer, Carlos Rossa, Angela C. Chi, Brad W. Neville, Tilman Heise

**Affiliations:** 1 Department of Biochemistry and Molecular Biology, College of Dental Medicine, Medical University of South Carolina, Charleston, South Carolina, United States of America; 2 Department of Craniofacial Biology, College of Dental Medicine, Medical University of South Carolina, Charleston, South Carolina, United States of America; 3 Department of Diagnosis and Surgery, Universidade Estadual Paulista (UNESP), Araraquara, Brazil; 4 Division of Oral Pathology, Medical University of South Carolina, Charleston, South Carolina, United States of America; University of Bergen, Norway

## Abstract

The 5-year survival rate for oral cavity cancer is poorer than for breast, colon or prostate cancer, and has improved only slightly in the last three decades. Hence, new therapeutic strategies are urgently needed. Here we demonstrate by tissue micro array analysis for the first time that RNA-binding protein La is significantly overexpressed in oral squamous cell carcinoma (SCC). Within this study we therefore addressed the question whether siRNA-mediated depletion of the La protein may interfere with known tumor-promoting characteristics of head and neck SCC cells. Our studies demonstrate that the La protein promotes cell proliferation, migration and invasion of lymph node-metastasized hypopharyngeal SCC cells. We also reveal that La is required for the expression of β-catenin as well as matrix metalloproteinase type 2 (MMP-2) within these cells. Taken together these data suggest a so far unknown function of the RNA-binding protein La in promoting tumor progression of head and neck SCC.

## Introduction

Head and neck (HN) cancer is the sixth most common cancer and affects more than 500,000 individuals globally [1], [2]. This type of cancer typically occurs in the mucosal lining of head or neck regions, e.g. esophagus, sinuses, nasal cavity, pharynx, mouth and lips. The most common type of HN cancer is the oral cavity cancer of which about 90% are squamous cell carcinomas (SCC) [3]. The 5-year survival rate for oral cavity cancer is 60%, which is poorer than that of breast, colon or prostate cancer, and only slightly improved by 5% in the last three decades [4]. Therefore, novel treatment strategies for oral SCC are urgently needed. Oral SCC grows locally in an invasive manner and mainly metastasizes to regional lymph nodes. Typically 50% of oral SCC patients have a detectable lymph node involvement at presentation. Patients with nodal metastasis have a markedly worse prognosis for treatment outcome [5]. The risk of regional metastasis is related to tumor size and depth of infiltration of the primary tumor [5]. The cellular and molecular mechanisms of tumor progression in the oral cavity remain poorly understood.

The La protein (La/SSB) is a ubiquitously and predominantly nuclear expressed RNA-binding protein, which is known to shuttle between the nucleus and cytoplasm [6], [7], [8], [9]. In mammals La is essential in early development [10]. La is a 408-amino acid protein with three RNA-binding surfaces (the La motif, and two RNA recognition motifs [11]), a nuclear localization sequence, a nuclear retention signal [12], and a nucleolus localization sequence [13]. Little is known about the regulation of La expression [8], [14]. La is posttranslationally modified by phosphorylation [6], [15], [16], [17] and sumoylation [18], both suggested regulating its cellular functions. La is presumed to act as a RNA chaperone to stabilize or refold RNA structures [19], [20]. La is known to promote processing of tRNA precursors and non-coding RNAs [12]. In addition, La supports mRNA translation of various cellular mRNAs, like cyclin D1 [21], XIAP [22] and Mdm2 [23]. Herein we present data demonstrating that the RNA-binding protein La is overexpressed in oral SCC tissue, and supports cell proliferation, migration and invasion of metastasis-derived hypopharyngeal SCC cells *in vitro,* as well as protein expression of tumor promoting factors β-catenin and MMP-2 but not cyclin D1, focal adhesion kinase (FAK) or cofilin within these cells. Our findings suggest that La may play an important role in tumor progression of head and neck SCC.

## Results and Discussion

### The RNA-binding protein La is overexpressed in oral SCC tissue

**Figure 1:**
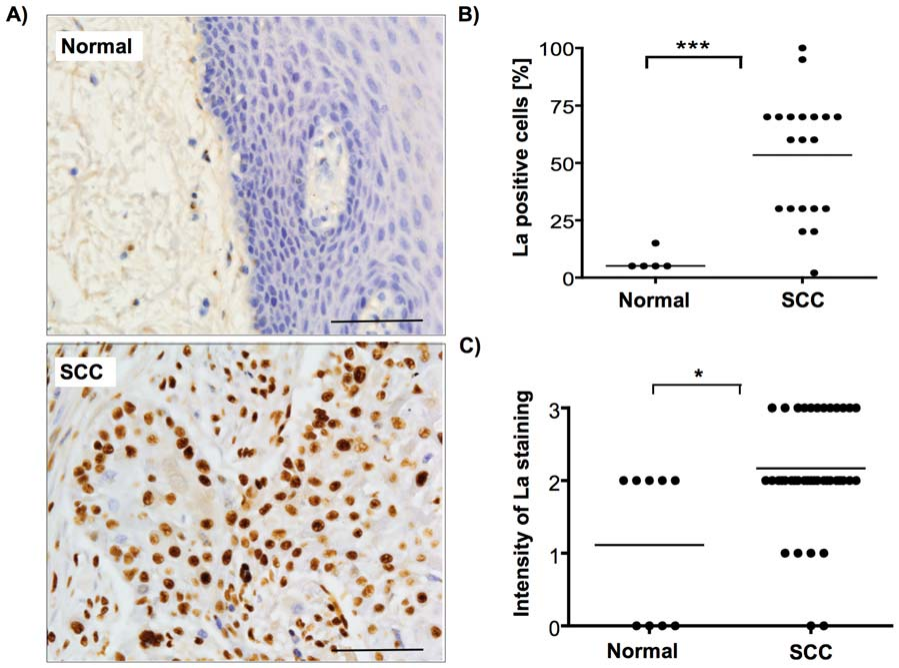
RNA-binding protein La is overexpressed in squamous cell carcinoma (SCC) tissue. **(A)** Human La-specific staining of normal epithelial and SCC tongue tissue by applying the monoclonal human La-specific 3B9 antibody. Scale bar represents 100 µm. **(B)** Scoring of oral tissue microarray (TMA) harboring 9 normal and 42 SCC tissue cores stained with human La-specific 3B9 antibody revealed highly significant overexpression of nuclear La in oral SCC. Percentage [%] of La-positive cells in normal versus SCC tissue (*P* value<0.001 (three asterisks)). **(C)** Intensity of nuclear La-staining in normal versus SCC tissue (*P* value<0.05 (one asterisks)). Two-tailed *P* values were determined by paired *t*-test applying Prism 4 (GraphPad Software).

Our previous studies revealed that the La protein is overexpressed in cervical cancer tissue [21]. To answer the question whether La is also aberrantly expressed in other types of cancer, we analyzed an oral cavity tissue microarray (TMA, Imgenex Corp., IMT-01250), consisting of 9 unmatched adjacent normal and 42 tumor tissue cores, diagnosed as SCC tissue ranging from grades I to III. La-specific immunohistochemical staining with monoclonal La antibody 3B9 [24] and counterstaining with Hematoxylin clearly revealed pronounced La-specific nuclear staining of SCC tissue but not normal epithelium (Fig. 1A). As a control, hybridization with immunoglobulin isotype IgG 2a, κ and counterstaining with Hematoxylin was performed, and no nonspecific brown staining was detected (Fig. S1). In addition we verified the specificity of the monoclonal human La 3B9 antibody by hybridization of siRNA-depleted UM-SCC 22B cells demonstrating that La-specific knock down within these cells almost completely abolishes the brown staining (Fig. S2). The TMA was scored by two pathologists independently and revealed that the percentage of La-positive cells was significantly higher in oral SCC tissue compared to normal epithelial tissue (Fig. 1B). In addition, the intensity of nuclear La staining was significantly higher in SCC cells compared to normal cells (Fig. 1C). These data show for the first time that not only more SCC cells express the La protein, but also that the La levels per cell were elevated. These data demonstrate that the RNA-binding protein La is aberrantly overexpressed in oral SCC tissue.

It has been shown that the La protein is overexpressed in a panel of tumor cell lines [25], chronic myelogenous leukaemia patients [23], and recently in cervical cancer tissue [21], suggesting a role of La in cancer pathogenesis. The finding that La stimulates mRNA translation of cyclin D1 in cervical cancer cells [21] and Mdm2 in BCR/ABL-transformed myeloid precursor cells [23], suggests an important role of La in translational control of tumor-promoting factors in cancerous cells. Although little is known about the regulation of La in cancerous cells, the finding that mammalian La is phosphorylated by Casein Kinase 2 [15], [16] and murine La by protein kinase AKT, which induces cytoplasmic accumulation of La and La-dependent translational activation of a specific mRNA subset [6], suggests that not only elevated La expression but also its phosphorylation status and/or intracellular localization is critical during tumorigenesis. However, our data are not allowing us to draw any conclusion about the intracellular localization of La in normal versus tumor cells and more work is needed to test whether the ratio of nuclear:cytoplasmic La is changed during tumor initiation or progression within the oral cavity. In conclusion we demonstrate that the RNA-binding protein La is significantly overexpressed in oral SCC tissue, suggesting that La may play a role in tumorigenesis of cancerous cells originating from various types of tissue [21], [23].

### The La protein contributes to proliferation and cell cycle progression of lymph node-metastasized hypopharyngeal SCC cells

**Figure 2:**
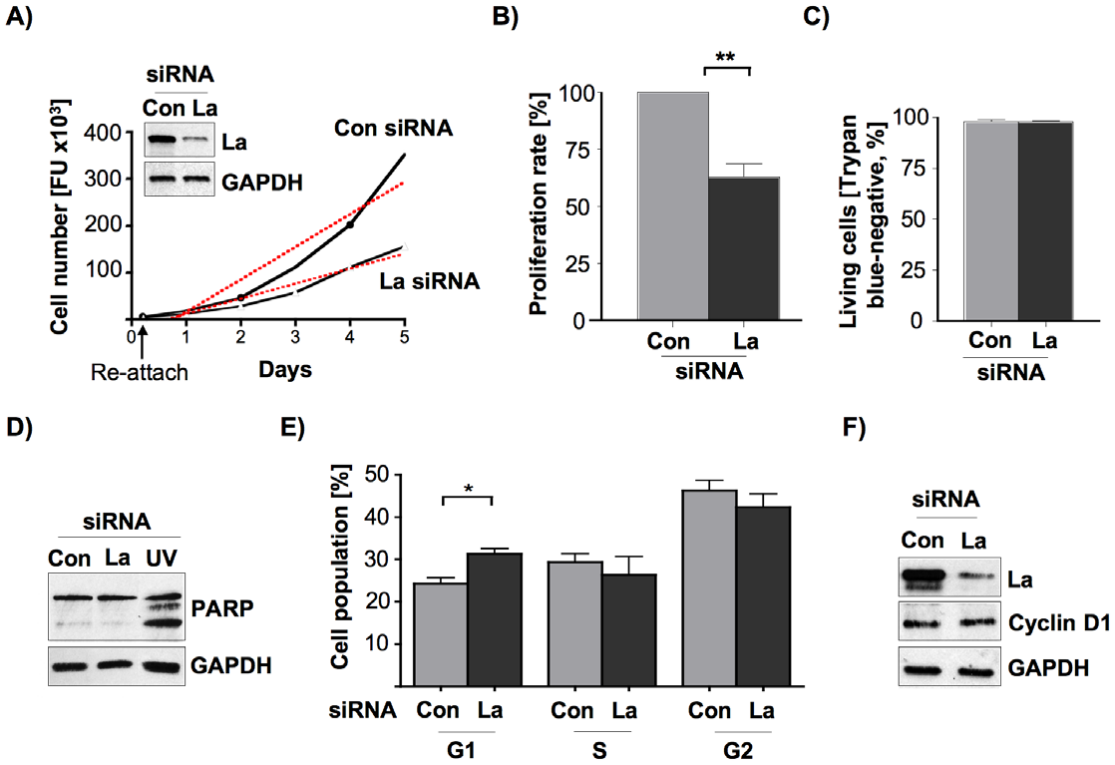
La protein expression promotes proliferation and cell cycle progression of SCC 22B cells. **(A)** Lower La expression in siRNA-mediated SCC 22B cells (see Western blot analysis in inlet) correlates with decreased proliferation rate (dotted line). **(B)** Reduced proliferation rate of La-depleted SCC 22B cells as determined as mean ±SD (error bars) of 5 independent experiments performed in triplicates. *P* value<0.01 (two asterisks). Two-tailed *P* value was determined by paired *t*-test applying Prism 4 (GraphPad Software). **(C)** SiRNA-mediated depletion of La does not reduce the number of viable cells as determined by counting of trypan blue-negative SCC 22B cells as determined as mean ±SD (error bars) of 3 independent experiments. **(D)** La depletion (La siRNA) in SCC 22B cells does not induce caspase-3 cleavage of PARP (under apoptosis PARP fragments into 116 and 85 kDa). Representative Western blot analysis of three independent experiments. As positive control PARP cleavage was induced by UV light (lane UV). GAPDH was applied as loading control. **(E)** SiRNA-mediated depletion of La correlates with a G1 cell cycle arrest in SCC 22B cells (*P* value<0.05 (one asterisks)). Two-tailed *P* value was determined by paired *t*-test applying Prism 4 (GraphPad Software). Error bars represent the mean ±SD of 3 independent experiments. **(F)** La depletion does not reduce cyclin D1 protein expression in SCC 22B cells. GAPDH was applied as loading control. Representative Western blot analysis of three independent experiments.

To elucidate whether La affects cellular processes known to contribute to tumorigenesis of HNSCC cells, we focused on UM-SCC 22B cells derived from a lymph node metastasis, which primary tumor site was presented as hypopharyngeal SCC [26] (from now on referred to as SCC 22B). To exclude a possible cross-contamination and overgrowth of cell line SCC 22B with HeLa cells, which were earlier cultured in our laboratory, we compared light microscopy images of our cultured SCC 22B cells with images taken from HeLa cells previously purchased from ATCC (Fig. S3). Morphological differences of the scale-like, polygonal SCC 22B cells and the rather elongated-shaped cervical cancer HeLa cells clearly distinguishes the two different cell lines by light microscopy. To test whether La protein regulates proliferation in metastasis-derived SCC cells, we reduced La levels in SCC 22B cells by transfecting La-specific small interfering RNA (La siRNA) [21] or luciferase-specific siRNA [27] as control (Con siRNA). Western blot analysis of cell lysates demonstrated the efficient depletion of La protein in siRNA-treated SCC 22B cells (Fig. 2A). The re-attachment efficiency was monitored after 5 hours by applying the CyQuant proliferation assay and showed that the re-attachment of the cells was unchanged despite La depletion (Fig. 2A). In contrast, the proliferation rate of La-depleted cells was significantly reduced, demonstrating that cells with reduced La expression grow about 40% slower compared to control-treated cells (Fig. 2B). To test whether La depletion induced cell death, the viability of La-depleted cells was analyzed by trypan blue exclusion assays (Fig. 2C), FACS analysis of Annexin V and propidium iodide co-stained cells (Fig. S4) and analysis of caspase-3-dependent PARP cleavage (Fig. 2D). All tests clearly demonstrated that the proliferation rate in La-depleted SCC 22B cells was not reduced by increased cell death, which is in line with studies previously performed in HeLa cells [21]. Next, cell cycle analysis of propidium iodide-stained cells by FACS showed that La depletion induced a G1-arrest in SCC 22B cells concomitant with a decrease of S- and G2-phase cell populations (Fig. 2E), suggesting that the proliferation rate was reduced via a defect in cell cycle progression. Since we have recently shown that overexpression of La stimulates and depletion of La reduces IRES-mediated cyclin D1 mRNA translation in cervical cancer HeLa cells [21], we analyzed whether the expression of the cell cycle regulator cyclin D1 is reduced in La-depleted SCC 22B cells as well. Our data clearly demonstrate that cyclin D1 expression in La-depleted SCC 22B cells is not affected (Fig. 2F). Hence, in contrast to cervical carcinoma cells in which La contributes to cyclin D1 expression and cell proliferation [21], La did not contribute to cyclin D1 expression in SCC 22B cells. Therefore, our data suggest that La regulates cyclin D1 expression in a tissue-specific manner. Taken together these data demonstrate that La expression contributes to cell proliferation and cell cycle progression of metastasis-derived SCC 22B cells.

### La supports SCC 22B cell migration *in vitro*

**Figure 3:**
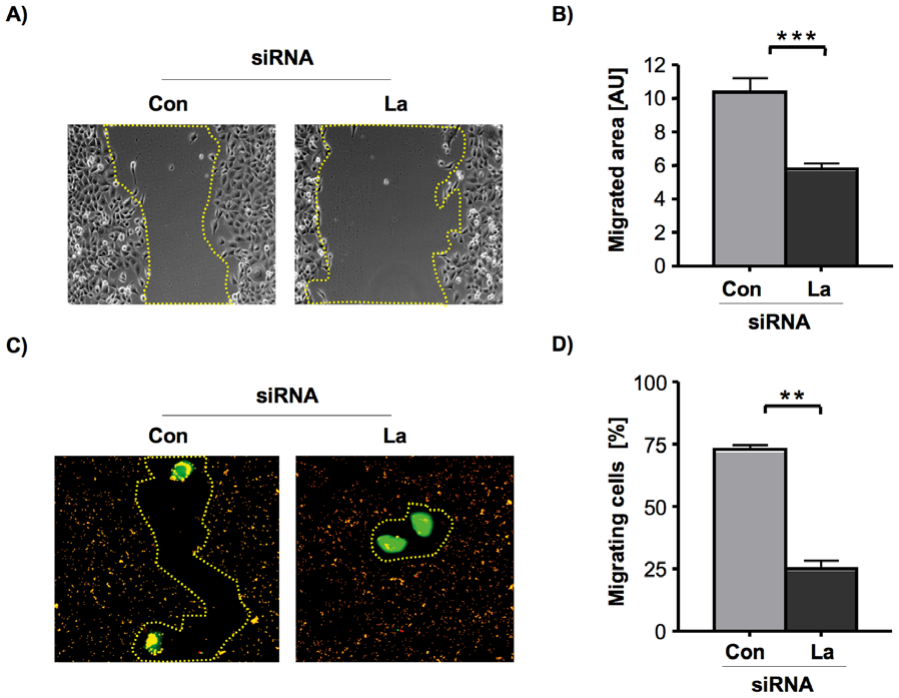
La supports cell motility of SCC 22B cells. **(A)** Wound healing assay: La depletion significantly reduces migration of UM-SCC 22B cells into the denuded area. Phase contrast pictures of the scratched area were taken at time 0 and after 24 hours using a Zeiss Axio Observer microscope and x10 objective power, and **(B)** the denuded area was quantified at each time point using NIH image software (AU = arbitrary units). Two-tailed *P* value<0.001 (three asterisks) was determined by paired *t*-test applying Prism 4 (GraphPad Software). Error bars represent the mean ±SD of 3 independent experiments in triplicates. **(C)** Migration track assay: On a field of fluorescent microspheres gfp-transfected La-depleted and control-treated SCC 22B cells were seeded at low density (4 cells per mm^2^) and non-fluorescent tracks created by gfp-transfected cells were evaluated by fluorescent microscopy after 24 hours using a Zeiss Axio Observer microscope and x40 objective power. **(D)** Quantification of cell motility by counting the number of non-fluorescent tracks of gfp-transfected cells ( = migrating cells). La depletion significantly reduces migration of single SCC 22B cells. Two-tailed *P* value<0.01 (two asterisks) was determined by paired *t*-test applying Prism 4 (GraphPad Software). Error bars represent the mean ±SD of 2 independent experiments in triplicates.

To address whether La is involved in tumor progression of SCC cells, we analyzed haptokinetic migration (movement of cells across extracellular matrix (ECM)) *in vitro,* applying two different cell migration assays. First we analyzed the effect of La depletion on migration of cell populations and performed wound-healing (scratch) assays [28], [29]. The ability of SCC 22B cells to migrate into a “wound” caused by a scratch was quantified by measuring the remaining open area of the scratch using NIH image after 24 hours (Fig. 3A). These studies demonstrate that La depletion reduced the repopulation of the wound by about 50% when compared with control-treated SCC 22B cells (Fig. 3B). Next we examined cell motility using the single-cell migration track assay. This assay examines the ability of individual cells to form migration tracks through a field of fluorescent microspheres [30]. We plated gfp-positive La-depleted or control-treated SCC 22B cells on fibronectin-coated plates covered with fluorescent microspheres. The next day, migrating cells were identified as cells that engulfed the fluorescent microspheres and left a track of non-fluorescence (Fig. 3C). Quantification of our data demonstrated that siRNA-mediated depletion of La reduces cell migration by about 65% when compared to control-treated cells (Fig. 3D). The results from both haptokinetic migration assays demonstrate that depletion of the RNA-binding protein La inhibits fundamental processes involved in cell migration of metastasis-derived 22B SCC cells, which is a so far unknown function for La and supports the notion that elevated La levels contribute to cell motility in HNSCC.

**Figure 4:**
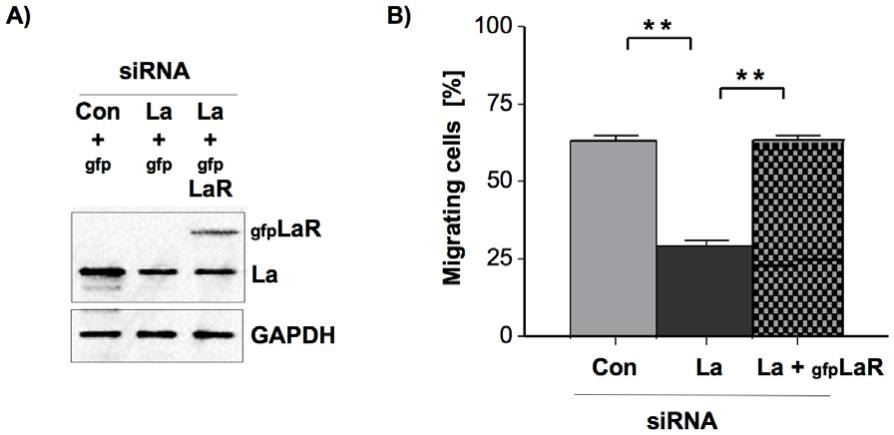
Rescue of reduced cell motility by overexpression of siRNA-resistant gfp-tagged La in La-depleted SCC 22B cells. (A) Western blot analysis demonstrates overexpression of gfp-tagged, La-siRNA resistant La in La-depleted (gfpLaR) SCC 22B cells. (D) Quantification of cell motility by counting the number of non-fluorescent tracks of gfp-transfected cells ( = migrating cells). La depletion significantly reduces migration of single SCC 22B cells and can be rescued to levels determined in control-treated cells by overexpression of La-siRNA resistant La. Two-tailed *P* value<0.01 (two asterisks) was determined by paired *t*-test applying Prism 4 (GraphPad Software). Error bars represent the mean ±SD of 2 independent experiments in triplicates.

To exclude “off-target” effects of the siRNA-mediated La depletion we performed siRNA rescue experiments. Therefore we transfected La-depleted 22B SCC cells with gfp-tagged La, which is resistant to La-specific siRNA due to three silent point mutations in the La siRNA binding site (gfpLaR) [21]. With these cells we determine whether the overexpression of siRNA-resistant La rescues the phenotype of reduced single-cell migration observed in La-depleted 22B SCC cells (Fig. 4A and B). Indeed, the data clearly show that the reduced migration of La-siRNA-treated 22B SCC cells was due to La-specific depletion of La and not due to unspecific off-target effects.

### Depletion of La reduces β-catenin protein expression in SCC 22B cells

**Figure 5:**
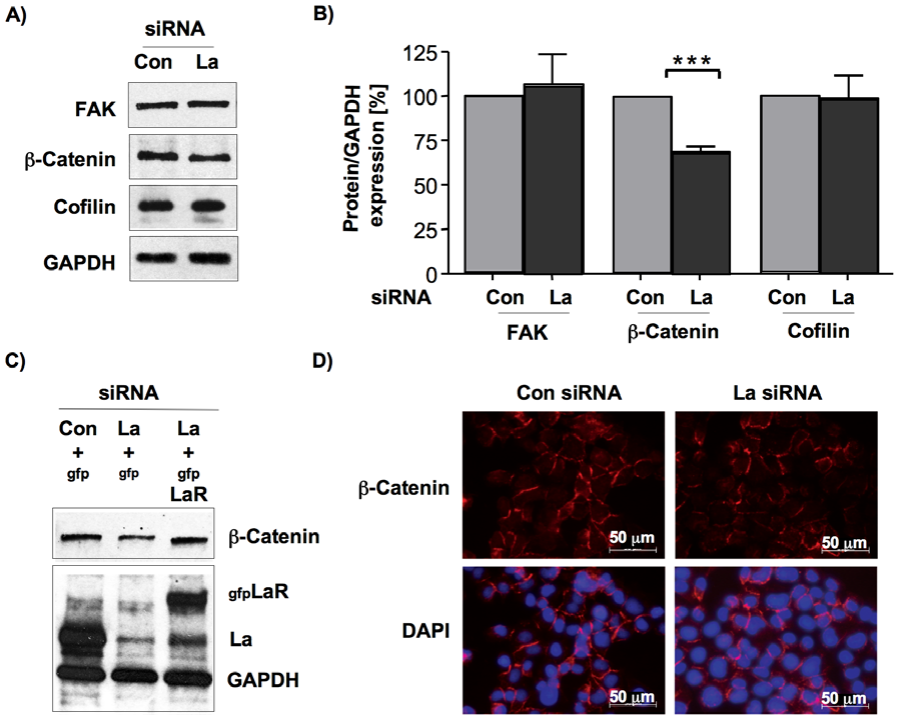
Reduced β-catenin protein expression in La-depleted SCC 22B cells. **(A)** Western blot analysis to determine FAK, β-catenin and cofilin protein expression levels in La-depleted SCC 22B cell extracts. GAPDH was applied as loading control. **(B)** For quantification of Western blot experiments chemiluminescent signals were recorded using an ImageQuant ECL systems and quantified using ImageQuant TL software. Two-tailed *P* value<0.001 (three asterisks) was determined by paired *t*-test applying Prism 4 (GraphPad Software). Error bars represent the mean ±SD of 4 independent experiments for β-catenin and 3 independent experiments for FAK and cofilin. **(C)** Overexpression of siRNA-resistant gfpLa protein (gfpLaR) rescues β-catenin expression in La-depleted SCC 22B cells. **(D)** Immunofluorescent staining shows localization of β-catenin within the cytoplasmic membrane in both La-depleted and control-treated SCC 22B cells.

To get insight into the role and function of La in cell motility we tested the expression of the following factors well known to be implicated in cell motility: focal adhesion kinase (FAK) [31], β-catenin [32] and cofilin [33]. Quantitative Western blot analysis and normalization to GAPDH revealed that the protein expression of β-catenin was significantly but only slightly reduced by 30% in La-depleted SCC 22B cells, whereas FAK and cofilin expression were unchanged (Fig. 5A and B). To demonstrate that the reduced β-catenin expression was not caused by siRNA-dependent off-target effects, we transfected La-depleted SCC 22B cells with the gfpLaR plasmid to overexpress siRNA-resistant gfpLa in cells in which endogenous La level were reduced due to La-specific siRNAs. Two days later we sorted the gfp-positive cells and performed Western blot analysis demonstrating that the expression of siRNA-resistant gfpLa protein (gfpLaR) rescues the β-catenin expression in La-depleted cells (Fig. 5C). This experiment underscores that β-catenin expression depends on cellular La protein level and is not caused by siRNA-dependent off-target effects.

Depending on its localization β-catenin plays a dual role within the cell: (I) in cytoplasmic membranes as part of the adherens junctions it regulates cell-cell adhesion [34], and (II) after Wnt-signaling activation β-catenin shuttles into the nucleus and up-regulates the transcription of tumor promoting target genes [35], [36]. In various types of cancer, including HNSCC, aberrant activation of β-catenin can promote tumor progression by positively effecting cell proliferation, differentiation, survival, adhesion, migration, invasion, and tumor growth in nude mice [36], [37], [38], [39], [40], [41], [42]. Reduced membranous staining for β-catenin was observed in less-differentiated oral SCC [43], [44] and increased β-catenin expression in HNSCC patient samples correlated with higher tumor stage [40], [45]. The localization of β-catenin to the cytoplasmic membrane was unchanged in La-depleted SCC cells (Fig. 5D). Therefore the reduced β-catenin protein level in La-depleted metastasis-derived SCC 22B cells suggests, that La supports β-catenin expression in metastatic SCC cells. Since it is known that the RNA-binding protein La stimulates mRNA translation in cancerous cells [21], [22], [23] and because cell migration depends on β-catenin mRNA translation in astrocytes [38], we speculate that elevated La stimulates β-catenin mRNA translation directly or stimulates a positive regulator of β-catenin expression in HNSCC.

### La expression promotes SCC 22B cell invasion and MMP-2 protein expression *in vitro*

**Figure 6:**
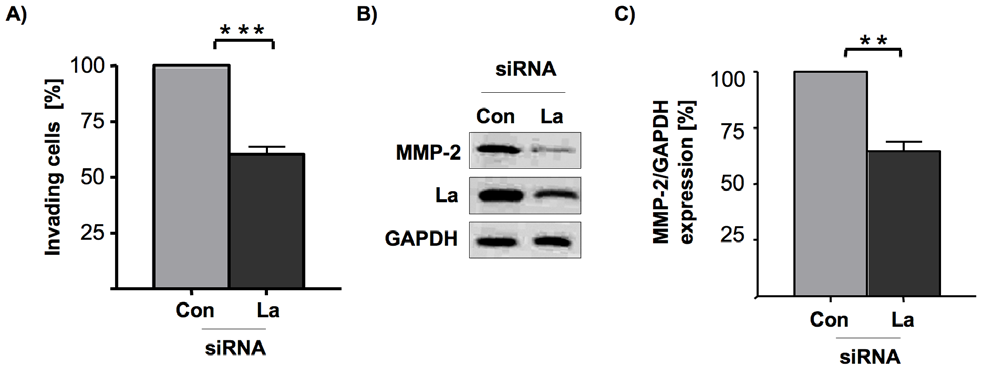
La promotes *in vitro* invasion and MMP-2 protein expression of SCC 22B cells. **(A)** *In vitro* invasion of La-depleted cells is reduced compared to control-treated SCC 22B cells. Two-tailed *P* value<0.001 (three asterisks) was determined by paired *t*-test applying Prism 4 (GraphPad Software). Error bars represent the mean ±SD of 3 independent experiments. **(B)** Reduced MMP-2 protein expression in La-depleted SCC 22B cells as determined by Western blot analysis. (C) For quantification of Western blot experiments chemiluminescent signals were recorded using an ImageQuant ECL systems and quantified using ImageQuant TL software. Two-tailed *P* value<0.01 (two asterisks) was determined by paired *t*-test applying Prism 4 (GraphPad Software). Error bars represent the mean ±SD of 4 independent experiments.

We also were interested to test whether La supports the invasive potential of metastasis-derived SCC cells. For those experiments we applied an *in vitro* invasion assay, in which we compared the ability of control-treated and La-depleted SCC cells to invade and move through Matrigel-coated transwells. As shown (Fig. 6A), the potential of La-depleted SCC cells to invade through the transwells was significantly diminished when compared to the control-treated cells. These results suggest that La affects both cell motility and invasiveness of metastasis-derived 22B SCC cells.

The ability of cells to invade requires the expression and activity of matrix metalloproteinases (MMPs), which are extracellular endopeptidases known to be key in breaking down barriers formed by the extracellular matrix [46], [47], [48]. MMP overexpression can facilitate tumor cell growth, migration, invasion, and metastasis in many different types of cancer, including oral cancer [49], [50]. In contrast, some MMPs exert dual functions and are able also to inhibit tumor progression depending on tumor stage, tumor site, enzyme localization and substrate, as recently reviewed in Gialeli et al. [51]. Interestingly, quantitative Western blot analysis revealed a strong reduction of MMP-2 protein level in La-depleted cells when compared with control-treated SCC 22B cells (Fig. 6B and C). Elevated MMP-2 expression in oral SCC correlates with aggressive oral tumor behavior and poor prognosis [52], [53], [54], [55] and was suggested as biomarker for early N-stage, aggressive oral SCC [53].

In summary these experiments illuminate a so far unexpected role of the RNA-binding protein La in cell motility and invasion. The finding that La promotes the expression of β-catenin and MMP-2 underscores an important role of La in tumor progression of HNSCC.

## Materials and Methods

### Cell lines, siRNA transfection and siRNA rescue

HNSCC cell line UM-SCC 22B [26] were kindly provided by Keith Kirkwood (MUSC) and grown in advanced DMEM plus 5% fetal bovine serum. La-specific depletion was performed as described recently [21], and shortly, by transfecting 2x10^6^ SCC 22B cells with 3 µg of La-specific (La siRNA) or control-siRNA (Con siRNA) using a Nucleofector according to the manufacturer's instructions (Nucleofector Kit V, program U-029, AMAXA, Germany). For gfp co-expression 2 µg of gfp expression plasmid (AMAXA) was added to the transfection mix. The siRNA duplexes were designed as 21-mers with 3′-dTdT overhangs [56] and synthesized by Dharmacon RNAi Technologies (USA). La-siRNAs were directed against the La sequence (NM_003142). La-siRNA (+): 5′-GAAACAGACCUGCUAAUACTT-3′ and La-siRNA (-) 3′-TTCUUUGUCUGGACGAUUAUG-5′; control-siRNAs are directed against the Luciferase sequence (referred to as Con [56]: Con-siRNA (+) 5′-CGUACGCGGAAUACUUCGATT-3′ and Con-siRNA (-) 3′-TTGCAUGCGCCUUAUGAAGCU-5′. For the rescue experiments applying PCR-based mutagenesis created the gfpLaR plasmid as described previously [21]. The following oligonucleotides, which introduce three silent point mutations (bold and underlined), were used: 5′-CCAGAAGTACAAAGAAAC**C**GA**T**CTGCT**G**ATACTTTTCAAGG-3′, 5′-CCTTGAAAAGTAT**C**AGCAG**A**TC**G**GTTTCTTTGTACTTCTGG-3′. For the rescue experiments 2x10^6^ La-depleted SCC 22B cells were transfected with 3 µg of gfpLaR or 2 µg of gfp expression plasmid (AMAXA) using a Nucleofector according to the manufacturer's instructions (Nucleofector Kit V, program U-029, AMAXA, Germany). La-depleted, control-treated and La-rescued SCC 22B cells were analyzed by Western blotting and single-cell migration track assays 24 hours after transfection.

### Immunohistochemistry and immunofluorescence

The immunohistochemical staining was performed as described recently [21] by applying the monoclonal human La-specific 3B9 antibody [200 ng/µl] [24] with a dilution of 1:200 on the oral cavity tissue microarray (TMA, Imgenex Corp., IMT-01250). For control staining mouse immunoglobulin isotype IgG2a,κ (eBioscience, [500 ng/µl], dilution 1:200) was applied on successive SCC tissue sections. For immunofluorescence the cells were fixed with 3.7% methanol-free formaldehyde (Thermo Scientific) for 10 min at room temperature. Afterwards cells were washed twice with 1x PBS and permeabilized with 0.3% Triton-X 100 in 1x PBS for 10 min at room temperature. Cells were washed five times with 1x PBS and blocked with horse serum (ImmPress Reagent Kit, Vector) for 30 min at room temperature. The anti-β-catenin antibody (#9581, Cell Signaling Technology, dilution 1:200) was applied over night at 4°C and thereafter washed off five times with 1x PBS. The secondary antibody (anti-rabbit Rhodamine RedX, Invitrogen, dilution 1:500) was applied for 30 min at room temperature. The cells were washed five times with 1x PBS and mounted with Vectashield Mounting Medium for Fluorescence with DAPI (Vector).

### Proliferation rate, Annexin V/PI analysis, cell cycle analysis and trypan blue exclusion assay

Proliferation rates were determined as described recently by applying the CyQuant Cell Proliferation Assay Kit (Invitrogen) [21]. The number of re-attached cells was determined 5 hours after seeding (FU = fluorescence units). The number of dead cells was determined by Annexin V and propidium iodide (PI) co-staining on day 2 after the second siRNA treatment applying the the Annexin-V-FLUOS Staining Kit (Roche). Annexin V staining and propidium iodide incorporation was determined by FACS analysis. For cell cycle analysis, cells were stained with propidium iodide two days after La-specific (La siRNA) and control (Con siRNA) siRNA treatment and cell cycle populations were analyzed by FACS, as described previously [21]. For the trypan blue exclusion assay trypsinized cells were stained with 0.2% trypan blue in 1x PBS followed by counting of unstained cells (viable) and stained cells (nonviable) in a hemocytometer.

### Western blot analysis

Cells were lysed and protein was analyzed by SDS-polyacrylamide gel electrophoresis (SDS-PAGE). The following antibodies were applied: monoclonal human La-specific 3B9 antibody [24], anti-PARP (#H-250, Santa Cruz, dilution 1:2,000), anti-Tubulin, (#T5168, Sigma, dilution 1:4,000), anti-GAPDH (#25778 Santa Cruz, dilution 1:2,000), anti-MMP-2 (#4022, Cell Signaling Technology, dilution 1:1,000), anti-cofilin (#C8736, Sigma, 1:1000), anti-β-catenin (#9581, Cell Signaling Technology, dilution 1:1,000), anti-FAK (#610087, Cell Signaling Technology, dilution 1:1,000). Secondary antibodies (dilution 1:20,000) were horseradish peroxidase-conjugated (Dianova, Germany).

### Wound healing assay

Wound healing assay was performed similar as described previously [29]. Briefly, La-depleted and control-treated UM-SCC 22B cells were allowed to grow to 90% confluence in a 12-well plate (Costar). The medium was replaced with serum-free medium and left overnight before scratching a wound using a 200 µl pipette tip. Phase contrast pictures of the scratched area were taken at time 0 and after 24 hours exactly at the same position at a marked area on the cell culture plate using a Zeiss Axio Observer microscope. The scratched area was quantified at each time point using NIH image software.

### Migration track assay

Wells were pre-coated with 5 µg/ml fibronectin (Fisher) in 1x PBS for 4 hours at room temperature or overnight at 4°C and then overlaid with a field of 1 µm in diameter carboxylate-modified polystyrene fluorescent microspheres (Invitrogen) in 1x PBS and left to settle for at least 2 hours at 4°C. Thereafter, gfp-transfected La-depleted and control-treated SCC 22B cells were seeded at low density (4 cells per mm^2^) in normal growth medium and incubated for a period of 24 hours. Non-fluorescent tracks created by gfp-transfected cells were then evaluated by fluorescent microscopy using a Zeiss Axio Observer microscope and quantified by counting the number of non-fluorescent tracks of gfp-transfected cells ( = migrating cells) on each plate.

### Invasion assay

*In vitro* invasion assays were performed according to the manufacturer's instructions (BD BioCoat Matrigel Invasion Chamber) or by coating 24-well Transwell plates (Corning) with 1mg/ml Matrigel (BD #354230) in 1x PBS. La-depleted or control-treated SCC 22B cells were plated in triplicates on Matrigel-coated Transwell inserts at 1×10^5^ in serum-free DMEM. The lower chamber contained DMEM with 5% FBS as a chemoattractant. After 24 hours of incubation, non-migrating cells were removed from the upper chamber with a cotton swab. According to the manufacturer's instructions (Cell Biolabs, colorimetric invasion assay, #CBA-100-C) cells that had invaded through the Matrigel-coated membrane to the lower surface were fixed, stained, extracted and quantified by optical density measurement at 560 nm applying a multiplate reader. Alternatively, cells on the lower surface of the membrane were fixed and stained according to manufacturer's instructions with the Diff-Quick Stain Kit (IMEBINC, #K7128) followed by mounting and counting cells within 10 random microscopic fields.

## Supporting Information

Figure S1 Control staining for La-specific immunohistochemistry in SCC tissue from oral cavity. Human La-specific antibody (anti-La 3B9) and control staining with mouse immunoglobulin isotype IgG2a,κ (IgG) of successive SCC tissue sections. Scale bar represents 100 µm.
(TIF)

Figure S2 SiRNA-mediated depletion of La demonstrates that anti-La 3B9 antibody specifically recognizes the RNA-binding protein La. Control siRNA- (Con) and La siRNA-treated SCC 22B cells were grown on glass slides, fixed with 3.7% formaldehyde and permeabilized with 0.3% Triton-X-100 in 1x PBS. La-specific brown staining was achieved following the protocol for IHC as described under MATERIAL/METHODS. Briefly, cells were blocked with mouse-serum (ImmPress Reagent, Vector), hybridized with anti-La 3B9 antibody (1:200), incubated with anti-mouse Ig peroxidase (ImmPress Reagent, Vector) and stained with ImmPACT DAB substrate (Vector). Counterstaining was performed with Hematoxylin QS (Vector). Scale bar represents 200 µm.
(TIF)

Figure S3 Morphological differences between SCC 22B and HeLa cells. Light microscopic images in low and high density of SCC 22B cells and HeLa cells purchased from ATCC. Scale bar represents 200 µm.
(TIF)

Figure S4 Depletion of La does not increase the number of dead SCC cells. The number of dead cells in La-depleted and control-treated SCC 22B cells was determined by Annexin V and propidium iodide (PI) co-staining. Error bars represent the mean ±SD from three independent experiments.
(TIF)

## References

[1] Parkin DM, Bray F, Ferlay J, Pisani P (2001). Estimating the world cancer burden: Globocan 2000.. Int J Cancer.

[2] Parkin DM, Bray F, Ferlay J, Pisani P (2005). Global cancer statistics, 2002.. CA Cancer J Clin.

[3] Beenken SW, Urist MM, Way LW, Doherty GM (2003). Current surgical diagnosis and treatment..

[4] Jemal A, Siegel R, Ward E, Hao Y, Xu J (2009). Cancer statistics.. CA Cancer J Clin.

[5] Sano D, Myers JN (2007). Metastasis of squamous cell carcinoma of the oral tongue.. Cancer Metastasis Rev.

[6] Brenet F, Socci ND, Sonenberg N, Holland EC (2009). Akt phosphorylation of La regulates specific mRNA translation in glial progenitors.. Oncogene.

[7] Fok V, Friend K, Steitz JA (2006). Epstein-Barr virus noncoding RNAs are confined to the nucleus, whereas their partner, the human La protein, undergoes nucleocytoplasmic shuttling.. J Cell Biol.

[8] Troster H, Metzger TE, Semsei I, Schwemmle M, Winterpacht A (1994). One gene, two transcripts: isolation of an alternative transcript encoding for the autoantigen La/SS-B from a cDNA library of a patient with primary Sjogrens' syndrome.. J Exp Med.

[9] Chambers JC, Kenan D, Martin BJ, Keene JD (1988). Genomic structure and amino acid sequence domains of the human La autoantigen.. J Biol Chem.

[10] Park JM, Kohn MJ, Bruinsma MW, Vech C, Intine RV (2006). The multifunctional RNA-binding protein La is required for mouse development and for the establishment of embryonic stem cells.. Mol Cell Biol.

[11] Kenan DJ, Keene JD (2004). La gets its wings.. Nat Struct Mol Biol.

[12] Wolin SL, Cedervall T (2002). The La protein.. Annu Rev Biochem.

[13] Horke S, Reumann K, Schweizer M, Will H, Heise T (2004). Nuclear trafficking of La protein depends on a newly identified nucleolar localization signal and the ability to bind RNA.. J Biol Chem.

[14] Carter MS, Sarnow P (2000). Distinct mRNAs that encode La autoantigen are differentially expressed and contain internal ribosome entry sites.. J Biol Chem.

[15] Schwartz EI, Intine RV, Maraia RJ (2004). CK2 is responsible for phosphorylation of human La protein serine-366 and can modulate rpL37 5′-terminal oligopyrimidine mRNA metabolism.. Mol Cell Biol.

[16] Intine RV, Sakulich AL, Koduru SB, Huang Y, Pierstorff E (2000). Control of transfer RNA maturation by phosphorylation of the human La antigen on serine 366.. Mol Cell.

[17] Broekhuis CH, Neubauer G, van der Heijden A, Mann M, Proud CG (2000). Detailed analysis of the phosphorylation of the human La (SS-B) autoantigen. (De)phosphorylation does not affect its subcellular distribution.. Biochemistry.

[18] van Niekerk EA, Willis DE, Chang JH, Reumann K, Heise T (2007). Sumoylation in axons triggers retrograde transport of the RNA-binding protein La.. Proc Natl Acad Sci U S A.

[19] Belisova A, Semrad K, Mayer O, Kocian G, Waigmann E (2005). RNA chaperone activity of protein components of human Ro RNPs.. RNA.

[20] Pannone BK, Xue D, Wolin SL (1998). A role for the yeast La protein in U6 snRNP assembly: evidence that the La protein is a molecular chaperone for RNA polymerase III transcripts.. EMBO J.

[21] Sommer G, Dittmann J, Kuehnert J, Reumann K, Schwartz PE (2011). The RNA-binding protein La contributes to cell proliferation and CCND1 expression.. Oncogene.

[22] Holcik M, Korneluk RG (2000). Functional characterization of the X-linked inhibitor of apoptosis (XIAP) internal ribosome entry site element: role of La autoantigen in XIAP translation.. Mol Cell Biol.

[23] Trotta R, Vignudelli T, Candini O, Intine RV, Pecorari L (2003). BCR/ABL activates mdm2 mRNA translation via the La antigen.. Cancer Cell.

[24] Kremerskothen J, Nettermann M, op de Bekke A, Bachmann M, Brosius J (1998). Identification of human autoantigen La/SS-B as BC1/BC200 RNA-binding protein.. DNA Cell Biol.

[25] Al-Ejeh F, Darby JM, Brown MP (2007). The La autoantigen is a malignancy-associated cell death target that is induced by DNA-damaging drugs.. Clin Cancer Res.

[26] Carey TE, Kimmel KA, Schwartz DR, Richter DE, Baker SR (1983). Antibodies to human squamous cell carcinoma.. Otolaryngol Head Neck Surg.

[27] Elbashir SM, Harborth J, Weber K, Tuschl T (2002). Analysis of gene function in somatic mammalian cells using small interfering RNAs.. Methods.

[28] Rodriguez LG, Wu X, Guan JL (2005). Wound-healing assay.. Methods Mol Biol.

[29] Liang CC, Park AY, Guan JL (2007). In vitro scratch assay: a convenient and inexpensive method for analysis of cell migration in vitro.. Nat Protoc.

[30] Windler-Hart SL, Chen KY, Chenn A (2005). A cell behavior screen: identification, sorting, and enrichment of cells based on motility.. BMC Cell Biol.

[31] Zhao J, Guan JL (2009). Signal transduction by focal adhesion kinase in cancer.. Cancer Metastasis Rev.

[32] Lai SL, Chien AJ, Moon RT (2009). Wnt/Fz signaling and the cytoskeleton: potential roles in tumorigenesis.. Cell Res.

[33] Bernstein BW, Bamburg JR (2010). ADF/cofilin: a functional node in cell biology.. Trends Cell Biol.

[34] Harris TJ, Tepass U (2010). Adherens junctions: from molecules to morphogenesis.. Nat Rev Mol Cell Biol.

[35] Heuberger J, Birchmeier W (2010). Interplay of cadherin-mediated cell adhesion and canonical Wnt signaling.. Cold Spring Harb Perspect Biol.

[36] Takemaru KI, Ohmitsu M, Li FQ (2008). An oncogenic hub: beta-catenin as a molecular target for cancer therapeutics..

[37] Yang F, Zeng Q, Yu G, Li S, Wang CY (2006). Wnt/beta-catenin signaling inhibits death receptor-mediated apoptosis and promotes invasive growth of HNSCC.. Cell Signal.

[38] Jones KJ, Korb E, Kundel MA, Kochanek AR, Kabraji S (2008). CPEB1 regulates beta-catenin mRNA translation and cell migration in astrocytes.. Glia.

[39] Iwai S, Yonekawa A, Harada C, Hamada M, Katagiri W (2010). Involvement of the Wnt-beta-catenin pathway in invasion and migration of oral squamous carcinoma cells.. Int J Oncol.

[40] Goto M, Mitra RS, Liu M, Lee J, Henson BS (2010). Rap1 stabilizes beta-catenin and enhances beta-catenin-dependent transcription and invasion in squamous cell carcinoma of the head and neck.. Clin Cancer Res.

[41] Inge LJ, Rajasekaran SA, Wolle D, Barwe SP, Ryazantsev S (2008). alpha-Catenin overrides Src-dependent activation of beta-catenin oncogenic signaling.. Mol Cancer Ther.

[42] Molinolo AA, Amornphimoltham P, Squarize CH, Castilho RM, Patel V (2009). Dysregulated molecular networks in head and neck carcinogenesis.. Oral Oncol.

[43] Lo Muzio L (2001). A possible role for the WNT-1 pathway in oral carcinogenesis.. Crit Rev Oral Biol Med.

[44] Ueda G, Sunakawa H, Nakamori K, Shinya T, Tsuhako W (2006). Aberrant expression of beta- and gamma-catenin is an independent prognostic marker in oral squamous cell carcinoma.. Int J Oral Maxillofac Surg.

[45] Yu Z, Weinberger PM, Provost E, Haffty BG, Sasaki C (2005). beta-Catenin functions mainly as an adhesion molecule in patients with squamous cell cancer of the head and neck.. Clin Cancer Res.

[46] Chen P, Parks WC (2009). Role of matrix metalloproteinases in epithelial migration.. J Cell Biochem.

[47] Deryugina EI, Quigley JP (2006). Matrix metalloproteinases and tumor metastasis.. Cancer Metastasis Rev.

[48] Deryugina EI, Quigley JP (2010). Pleiotropic roles of matrix metalloproteinases in tumor angiogenesis: contrasting, overlapping and compensatory functions.. Biochim Biophys Acta.

[49] Kessenbrock K, Plaks V, Werb Z (2010). Matrix metalloproteinases: regulators of the tumor microenvironment.. Cell.

[50] Rosenthal EL, Matrisian LM (2006). Matrix metalloproteases in head and neck cancer.. Head Neck.

[51] Gialeli C, Theocharis AD, Karamanos NK (2011). Roles of matrix metalloproteinases in cancer progression and their pharmacological targeting.. FEBS J.

[52] Gao ZB, Duan YQ, Zhang L, Chen DW, Ding PT (2005). Expression of matrix metalloproteinase 2 and its tissue inhibitor in oral squamous cell carcinoma.. Int J Mol Med.

[53] Mitra RS, Goto M, Lee JS, Maldonado D, Taylor JM (2008). Rap1GAP promotes invasion via induction of matrix metalloproteinase 9 secretion, which is associated with poor survival in low N-stage squamous cell carcinoma.. Cancer Res.

[54] Patel BP, Shah SV, Shukla SN, Shah PM, Patel PS (2007). Clinical significance of MMP-2 and MMP-9 in patients with oral cancer.. Head Neck.

[55] Ruokolainen H, Paakko P, Turpeenniemi-Hujanen T (2006). Tissue and circulating immunoreactive protein for MMP-2 and TIMP-2 in head and neck squamous cell carcinoma–tissue immunoreactivity predicts aggressive clinical course.. Mod Pathol.

[56] Elbashir SM, Harborth J, Lendeckel W, Yalcin A, Weber K (2001). Duplexes of 21-nucleotide RNAs mediate RNA interference in cultured mammalian cells.. Nature.

