# Supplementary figures and images for: Implication of RNA-Binding Protein La in Proliferation, Migration and Invasion of Lymph Node-Metastasized Hypopharyngeal SCC Cells
Source: PLoS One. 2011 Oct 10;6(10):e25402. doi: 10.1371/journal.pone.0025402 (PMC3189910; doi:10.1371/journal.pone.0025402)

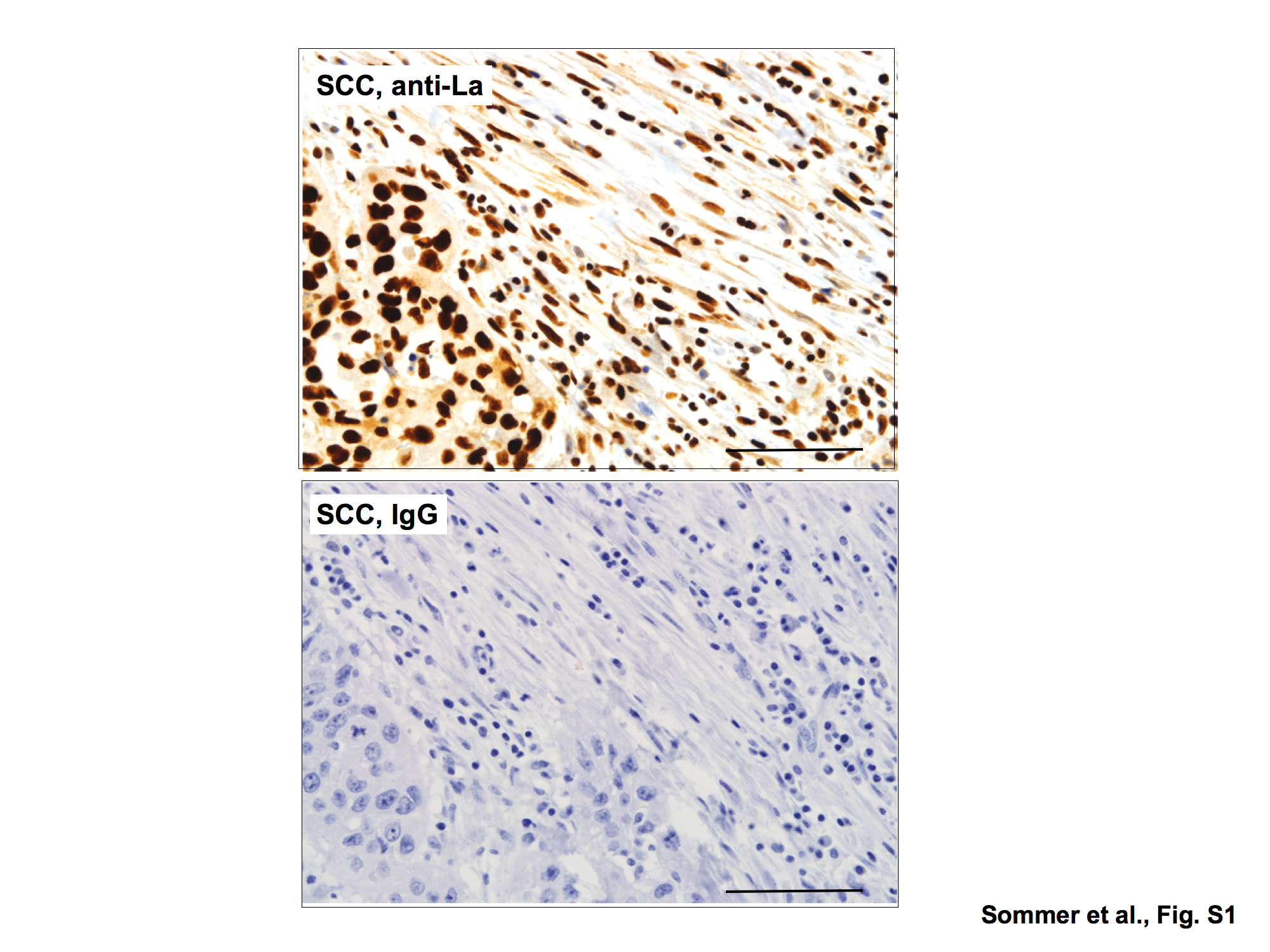

Supplement: Figure S1 — Control staining for La-specific immunohistochemistry in SCC tissue from oral cavity. Human La-specific antibody (anti-La 3B9) and control staining with mouse immunoglobulin isotype IgG2a,κ (IgG) of successive SCC tissue sections. Scale bar represents 100 µm. (TIF) [file pone.0025402.s001.tif]

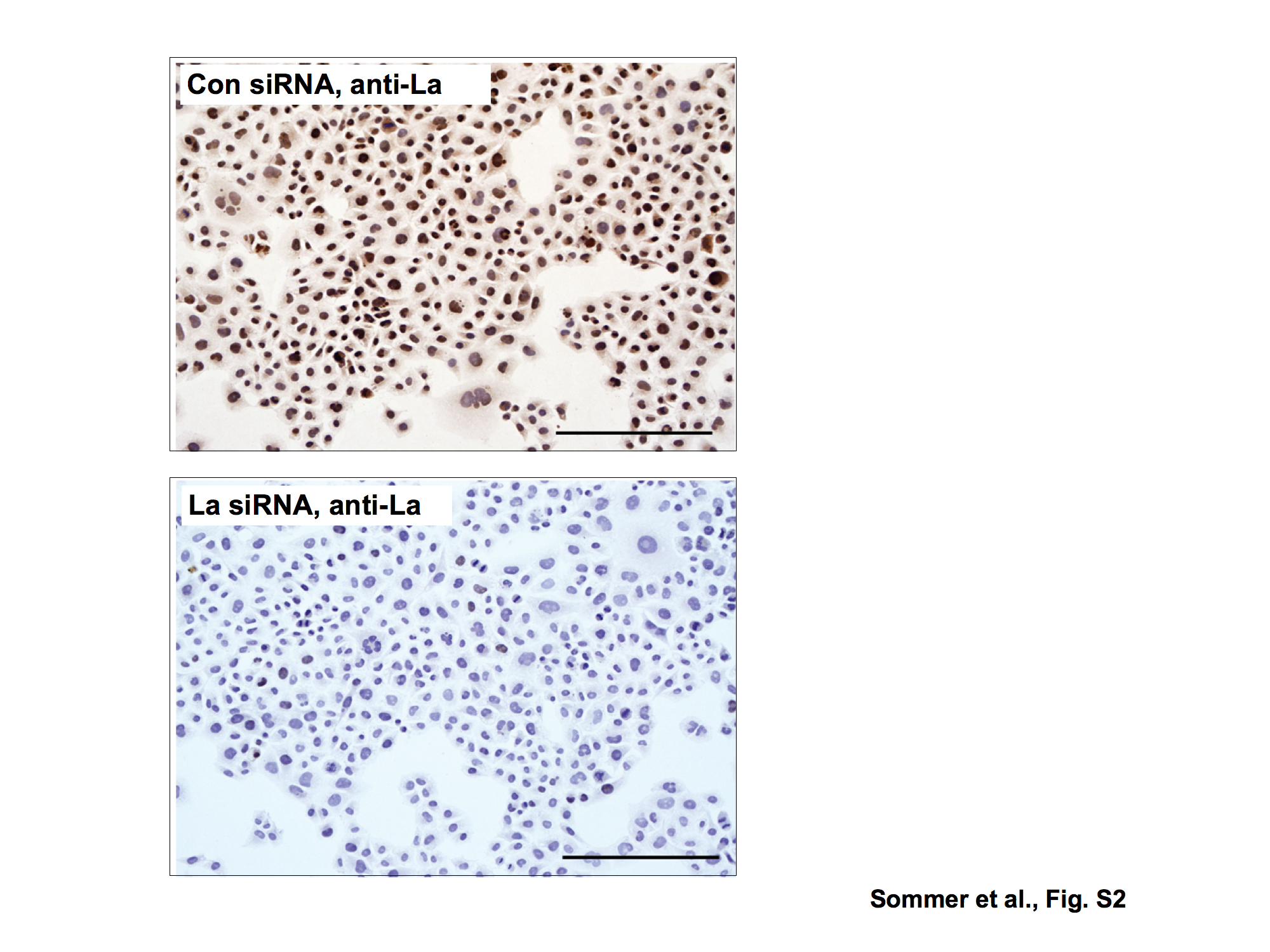

Supplement: Figure S2 — SiRNA-mediated depletion of La demonstrates that anti-La 3B9 antibody specifically recognizes the RNA-binding protein La. Control siRNA- (Con) and La siRNA-treated SCC 22B cells were grown on glass slides, fixed with 3.7% formaldehyde and permeabilized with 0.3% Triton-X-100 in 1x PBS. La-specific brown staining was achieved following the protocol for IHC as described under MATERIAL/METHODS. Briefly, cells were blocked with mouse-serum (ImmPress Reagent, Vector), hybridized with anti-La 3B9 antibody (1:200), incubated with anti-mouse Ig peroxidase (ImmPress Reagent, Vector) and stained with ImmPACT DAB substrate (Vector). Counterstaining was performed with Hematoxylin QS (Vector). Scale bar represents 200 µm. (TIF) [file pone.0025402.s002.tif]

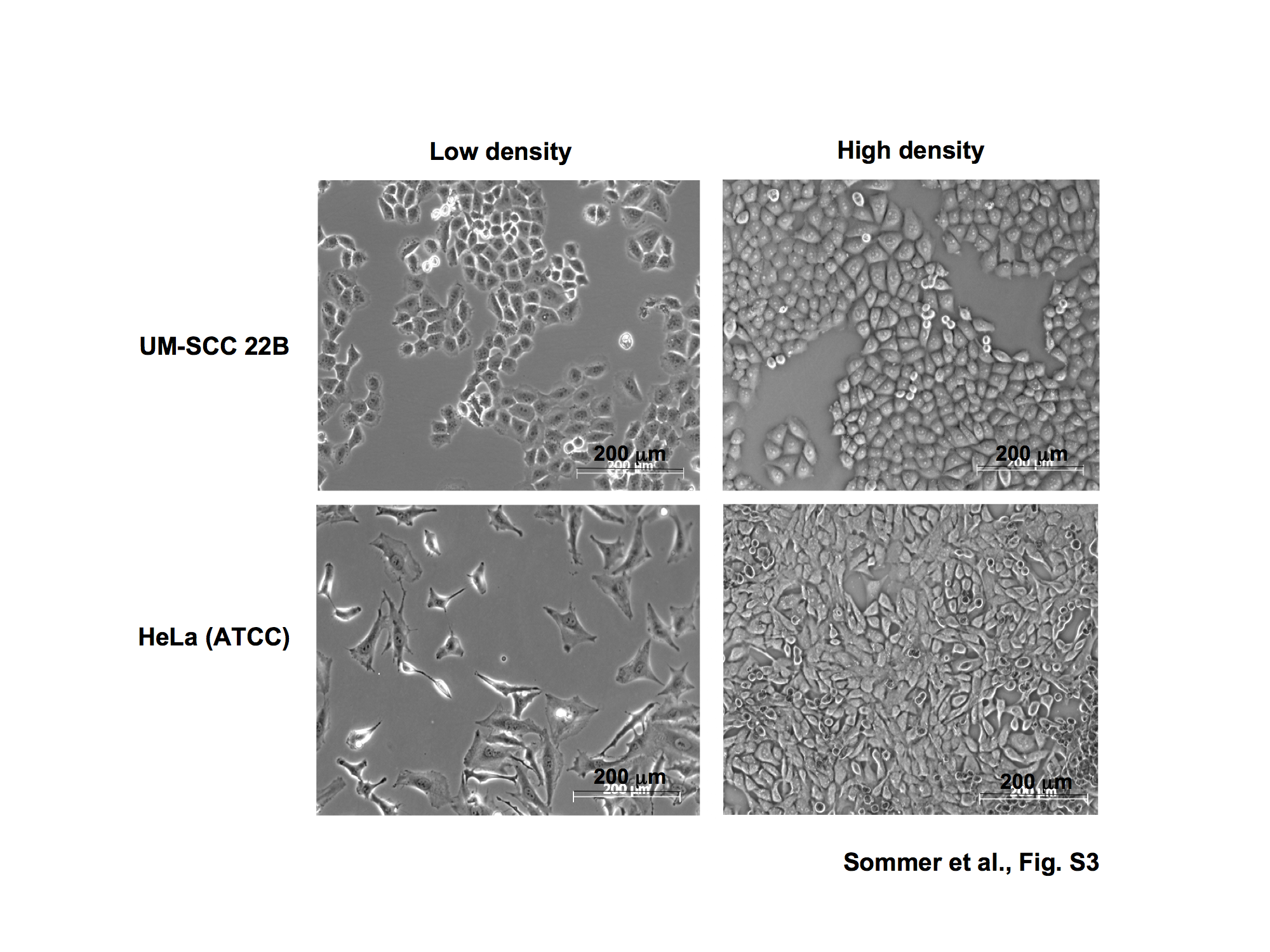

Supplement: Figure S3 — Morphological differences between SCC 22B and HeLa cells. Light microscopic images in low and high density of SCC 22B cells and HeLa cells purchased from ATCC. Scale bar represents 200 µm. (TIF) [file pone.0025402.s003.tif]

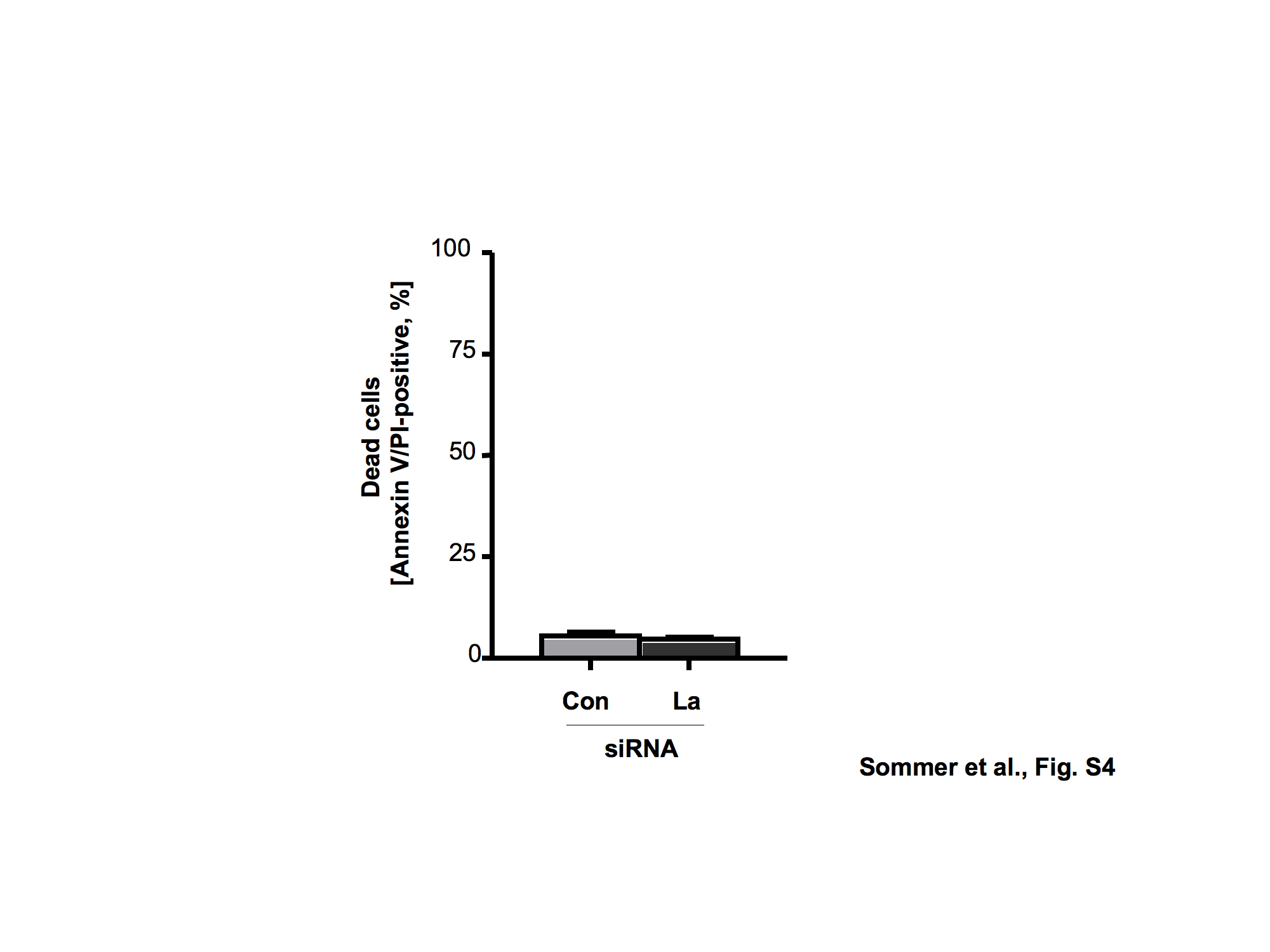

Supplement: Figure S4 — Depletion of La does not increase the number of dead SCC cells. The number of dead cells in La-depleted and control-treated SCC 22B cells was determined by Annexin V and propidium iodide (PI) co-staining. Error bars represent the mean ±SD from three independent experiments. (TIF) [file pone.0025402.s004.tif]
